# Palmer amaranth's arsenal: Rearrangement of eccDNA provides dual herbicide resistance in *Amaranthus palmeri*

**DOI:** 10.1093/plcell/koaf077

**Published:** 2025-04-02

**Authors:** Yu-Hung Hung

**Affiliations:** Assistant Features Editor, The Plant Cell, American Society of Plant Biologists; Donald Danforth Plant Science Center, St. Louis, MO 63132, USA

“Pigweed” is a simple word that can strike fear in the heart of many farmers. *Amaranthus palmeri*, also known as Palmer amaranth, is one of the most difficult pigweeds to control, thriving in cotton, corn, and soybean fields and outcompeting the crops for nutrients, water, and sunlight. This monster-sized weed is fast-growing, massively reproductive, and can cause up to 90% yield loss throughout the United States in certain row crops (Massinga et al. 2001).

Glyphosate, the active ingredient in RoundUp, was introduced in the 1970s. At first, it was only good for bare-ground type applications when control of all green plants was desired. However, the introduction of RoundUp Ready crops in the 1990s led to it being the most commonly used herbicide in row crops in the US. Palmer amaranth was kept at bay by broadcast application of the RoundUp brand of glyphosate. However, after about 10 years, some populations of Palmer amaranth began to evolve resistance to glyphosate (Culpepper et al. 2006). How did this happen?

Scientists who studied these glyphosate-resistant Palmer amaranth accessions discovered that the plant's DNA contains large, self-replicating extra-chromosomal circular DNA (eccDNA) in addition to the normal chromosomes (Koo et al. 2018; Molin et al. 2020). The eccDNA harbors the *5-enolpyruvylshikimate-3-phosphate synthase* (*EPSPS*) gene, which encodes for the EPSPS protein that glyphosate targets. The *EPSPS* gene in eccDNA is from its own genome in Palmer amaranth. Susceptible Palmer amaranth has only 1 copy of *EPSPS* in its genome, and therefore the base level of EPSPS protein in susceptible plants is sufficiently inhibited by field rates of glyphosate. However, the eccDNA-containing Palmer amaranth can survive glyphosate treatment because it has dozens or even hundreds of eccDNA copies, each contributing to a greatly increased EPSPS protein pool. The amount of glyphosate needed to inhibit EPSPS is directly correlated with the size of the EPSPS protein pool. Therefore, by retaining enough copies of *EPSPS*, the Palmer amaranth becomes resistant to typical field doses of glyphosate treatment (Gaines et al. 2010).

One of the reasons that Palmer amaranth became the most problematic weed in the US is its ability to rapidly evolve herbicide resistance. Rampant glyphosate resistance has forced farmers to use alternative herbicides to fight back Palmer pigweed. With the commercialization of glufosinate-resistant crops, glufosinate-ammonium became an important alternative herbicide for Palmer amaranth control. This herbicide inhibits the enzyme glutamine synthetase (GS), which has 2 isoforms in plants: *GS1* and *GS2* (McNally et al. 1983). Not surprisingly, Palmer amaranth has begun evolving resistance after recurrent application of glufosinate-ammonium (Carvalho-Moore et al. 2022; Noguera et al. 2022; Priess et al. 2022). Furthermore, previous studies have revealed *GS2* amplification in Palmer amaranth, similar to what was seen 20 years ago with glyphosate (Carvalho-Moore et al. 2022; Noguera et al. 2022). However, the mechanisms of glufosinate-ammonium resistance are still under investigation.

In their new work, Carvalho-Moore and colleagues (Carvalho-Moore et al. 2025) used PacBio HiFi long read resequencing, digital PCR, and molecular markers to uncover the specific genomic structural variant responsible for *GS2* amplification. The authors discovered that some of the eccDNAs in an *A. palmeri* accession (MSR2) resistant to both glyphosate and glufosinate-ammonium now also included the *GS2* gene beside the original *EPSPS* gene. The novel eccDNA is about 26 kb longer than the previously identified eccDNA in *A. palmeri*. The 2 eccDNAs share high similarity and synteny, but a region in the original eccDNA has been replaced with a locus containing tandemly duplicated *GS2* isoforms along with other genes in the genome (Figure).

**Figure.**
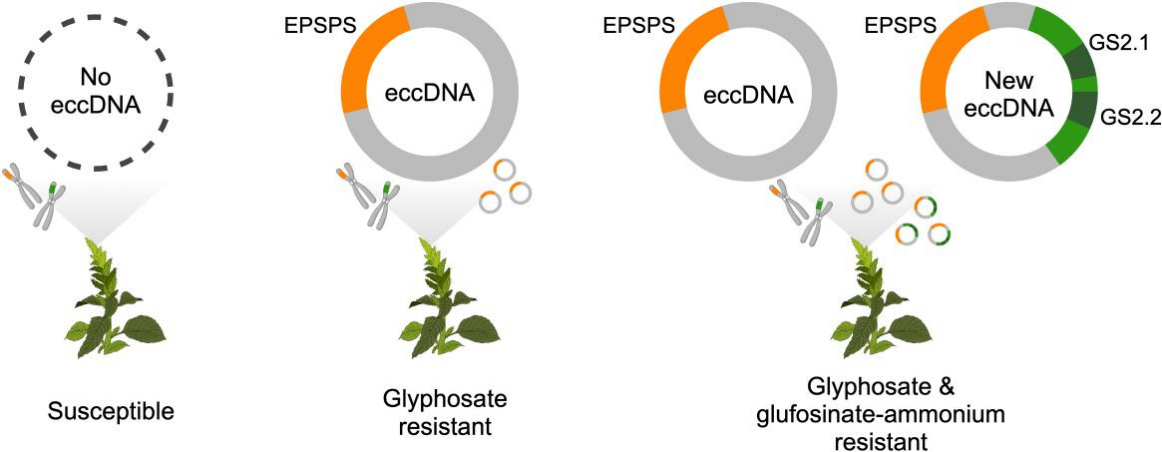
Palmer amaranth's arsenal. Susceptible Palmer pigweed contains no eccDNA. Glyphosate-resistant plants contain eccDNA with the *EPSPS* gene. In a new study, a novel eccDNA containing both *EPSPS* and 2 *GS2* isoforms, *GS2.1* and *GS2.2*, confers resistance to both glyphosate and glufosinate-ammonium. Figure created by Y-H. Hung using BioRender.

In addition, the authors found that both the original eccDNA (with only *EPSPS*) and the newly discovered eccDNA (with *EPSPS* and *GS2.1*/*GS2.2*) were present in the same individual plant, indicating that both eccDNAs coexist and independently replicate (Figure). MSR2 plants carrying both versions of eccDNA have 6 times the sequencing read depth for the *EPSPS* region compared with plants carrying eccDNA with only *EPSPS*. This indicates an additive copy number amplification for *EPSPS* between the 2 eccDNA replicons.

A second *A. palmeri* accession called MSR1, also resistant to glyphosate and glufosinate-ammonium, was used to further investigate *GS2* gene amplification. Comparison of the copy number of *GS2* isoforms between MSR1 and MSR2 accessions showed different patterns of *GS2* amplification. The MSR1 showed amplification of *EPSPS* and *GS2.1* only, and their copy number amount was independent/unlinked, while the MSR2 showed amplification of *EPSPS* and both *GS2.1* and *GS2.2* isoforms and that the *GS2* and *EPSPS* genes were physically linked. This result suggested the presence of a third yet-to-be-identified eccDNA containing only *GS2.2* in MSR2.

The discovery of this eccDNA variant in *A. palmeri* that confers dual resistance to glyphosate and glufosinate-ammonium highlights the remarkable adaptability of Palmer pigweed. This finding also unveils that Palmer amaranth can expand its arsenal against different herbicides through eccDNA. While herbicide resistance is an easily observed phenotype and therefore one that can be studied, eccDNAs may exist for many other important traits that are harder to detect. Circular DNA is found across all kingdoms and in some tissues like cancer, suggesting that it plays a fundamental role in cells and can alter cell and organism phenotype for many traits (Noer et al. 2022). Further research into the genetics and molecular components of eccDNAs in pigweed could provide models for developing more effective weed management strategies or mechanisms to reverse herbicide resistance.

## Recent related articles in *The Plant Cell*

- Molin et al. (2020) discovered a massive extrachromosomal circular DNA that harbors the *EPSPS* gene and 58 other genes whose encoded functions traverse detoxification, replication, recombination, transposition, tethering, and transport in *A. palmeri*.
- Paterson and Queitsch (2024) reviewed the interplay between angiosperm genome organization and botanical diversity.
- Yaschenko et al. (2024) highlighted the utility and challenges of using Arabidopsis as a reference for applied plant biology research, including herbicide resistance studies.
